# The Bile Acid Synthesis Pathway Is Present and Functional in the Human Ovary

**DOI:** 10.1371/journal.pone.0007333

**Published:** 2009-10-06

**Authors:** Laura P. Smith, Maik Nierstenhoefer, Sang Wook Yoo, Alan S. Penzias, Edda Tobiasch, Anny Usheva

**Affiliations:** 1 Division of Reproductive Endocrinology & Infertility, Department of Obstetrics & Gynecology, Beth Israel Deaconess Medical Center and Harvard Medical School, Boston, Massachusetts, United States of America; 2 Division of Endocrinology, Department of Medicine, Beth Israel Deaconess Medical Center and Harvard Medical School, Boston, Massachusetts, United States of America; 3 Boston IVF, Waltham, Massachusetts, United States of America; 4 University of Applied Sciences Bonn-Rhein-Sieg, Rheinbach, Germany; Sun Yat-Sen University, China

## Abstract

**Background:**

Bile acids, end products of the pathway for cholesterol elimination, are required for dietary lipid and fat-soluble vitamin absorption and maintain the balance between cholesterol synthesis in the liver and cholesterol excretion. They are composed of a steroid structure and are primarily made in the liver by the oxidation of cholesterol. Cholesterol is also highly abundant in the human ovarian follicle, where it is used in the formation of the sex steroids.

**Methodology/Principal Findings:**

Here we describe for the first time evidence that all aspects of the bile acid synthesis pathway are present in the human ovarian follicle, including the enzymes in both the classical and alternative pathways, the nuclear receptors known to regulate the pathway, and the end product bile acids. Furthermore, we provide functional evidence that bile acids are produced by the human follicular granulosa cells in response to cholesterol presence in the culture media.

**Conclusions/Significance:**

These findings establish a novel pathway present in the human ovarian follicle that has the capacity to compete directly with sex steroid synthesis.

## Introduction

Bile acids are powerful detergents with essential functions in lipid and cholesterol processing. They play key roles in the gastrointestinal and hepatobiliary systems, are required for dietary lipid and fat-soluble vitamin absorption, and maintain the balance between cholesterol synthesis in the liver and cholesterol excretion [1]–[3]. Bile acids are composed of a steroid structure and are made primarily in the liver by the oxidation of cholesterol. The synthesis of bile acids and their localization are very tightly regulated due to their membrane and epithelial toxicity [3], [4].

Bile acid synthesis can occur through two pathways: the classic (neutral) pathway or the alternative (acidic) pathway [5]. The classic pathway occurs in the liver, and accounts for approximately 90% of bile acid synthesis. The alternative pathway produces the remaining 10% of bile acids and is primarily extrahepatic. The classical pathway begins with the rate-limiting 7-α hydroxylation of cholesterol by cholesterol 7-α hydroxylase (CYP7A1) and the 12-α hydroxylation of the intermediates by sterol 12-α hydroxylase (CYP8B1), followed by side chain oxidation by sterol 27 hydroxylase (CYP27A1) [2], [6]–[8]. The alternative pathway begins with the hydroxylation of the cholesterol side chain by sterol 27 hydroxylase (CYP27A1) in extrahepatic sites including vascular endothelium and macrophages, followed by 7-α hydroxylation of the oxysterol intermediates by oxysterol 7-α hydroxylase (CYP7B1) [6], [9].

There are well-known feedback loops controlling gene transcription of the major enzymes in bile acid synthesis, especially CYP7A1 and CYP8B1 [6], [7], [10]–[12]. The nuclear receptors that have been demonstrated to regulate the bile acid synthesis cascade include the Farnesoid X Receptor (FXR), Retinoid X Receptor α (RXRα), Liver X Receptor α (LXRα), and Liver Receptor Homologue 1 (LRH-1) [6], [13]–[15]. Only LRH-1 has been previously studied in steroidogenic tissue, including ovaries, from mice and rats [16]–[18].

In addition to its presence in the gastrointestinal and hepatobiliary systems, cholesterol is known to be highly abundant in the human ovarian follicle, where it is used in the formation of the sex steroids [19]–[21]. The human ovarian follicle is the fluid-filled sac that contains the oocyte (egg), and is lined with granulosa cells which house the enzymatic machinery to participate in sex steroid synthesis. Given the high content of both cholesterol and the sex steroids in the human ovarian follicles, we searched for the presence of bile acids in the ovarian follicular fluid [22].

Here we describe for the first time evidence that all aspects of the bile acid synthesis pathway are present in the human ovarian follicle, including the enzymes in both the classical and alternative pathways, the nuclear receptors known to regulate the pathway, and the end product bile acids. Furthermore, we provide functional evidence that bile acids are produced by the human follicular granulosa cells in response to cholesterol presence in the culture media. These findings establish a novel pathway present in the human ovarian follicle that has the capacity to compete directly with sex steroid synthesis.

## Results

### Bile Acids are Present in Human Ovarian Follicular Fluid

**Figure 1:**
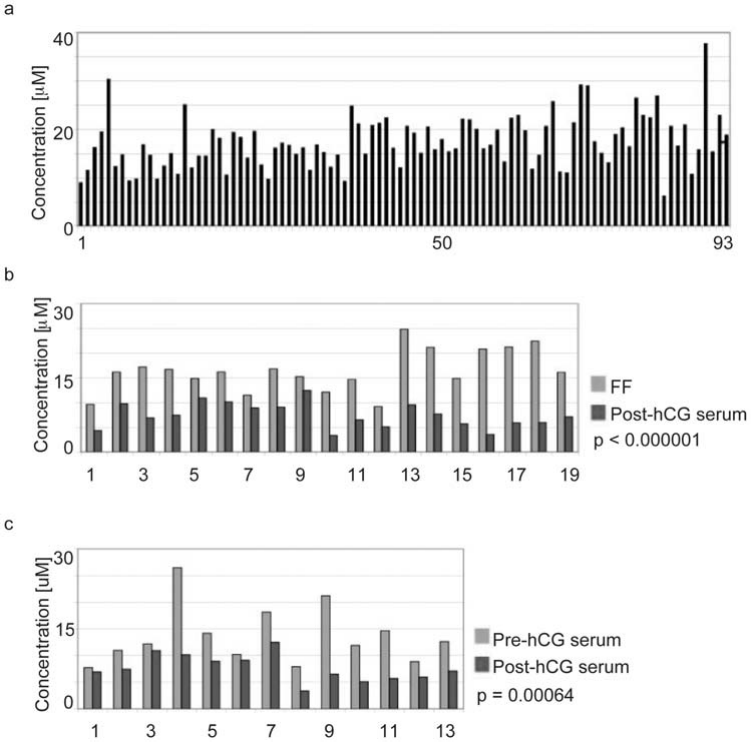
Presence of bile acids in human ovarian follicular fluid. Bile acid concentration was measured in FF (Panel a), in FF compared to post-HCG serum (Panel b), and in pre-HCG serum compared to post-HCG serum (Panel c).

Follicular fluid (FF) from mature follicles from 93 patients was obtained during *in vitro* fertilization (IVF). The individual FF samples were analyzed in duplicate for content of total bile acids by enzymatic assay. The mean bile acid concentration in FF was measured to be 17.301 µM (STD ± 5.41) (Figure 1, a). The origin of bile acids in FF is not known. They could enter the follicle through the blood, or they could be synthesized in the follicular compartment. In the case of diffusion from blood, their content in FF and serum is expected to be nearly identical. Alternately, active bile acid synthesis within the follicle could result in a higher follicular content compared to serum. In order to investigate the bile acids origin we compared content in blood and FF in 19 of the patients. The mean concentration of bile acids in serum obtained within 30 minutes of FF collection was found to be 7.672 µM (STD ± 2.548) (Figure 1, b). This dramatic difference of bile acid content in FF and serum is surprising and statistically significant (p<0.000001). The IVF procedure includes treatment with human chorionic gonadotropin (hCG). It is likely that the observed difference is a result of the hCG treatment. Therefore, we compared bile acid content in blood collected 24 hours before and 36 hours after hCG administration in 13 of these patients to evaluate the impact of hCG administration on bile acid content (Figure 1, c). The mean pre-hCG bile acid concentration in the serum of the 13 samples was measured to be higher (13.61 µM, STD ± 5.47) than in the post-hCG serum (7.672 µM) (p = 0.00064). The mean pre-hCG bile acid concentration in the serum (13.61 µM ) was lower than in the corresponding FF (17.301 µM) but this difference was not statistically significant (p = 0.185). The highly significant difference in the bile acid content between FF and the post-hCG serum obtained within 30 minutes of FF collection supports the proposal for active intrafollicular ovarian synthesis.

This is the first demonstration of the presence of bile acids in this compartment. The high cholesterol concentration in the follicle is likely to be supporting not only active steroidogenesis but also synthesis of bile acids. This finding raises the obvious question of whether the enzymes responsible for bile acid synthesis are present in the ovarian follicle. The intrafollicular synthesis of bile acids could be facilitated by activities within the granulosa cells, the oocyte, or both.

### Enzymes in the Bile Acid Synthesis Pathway are Present in the Ovarian Follicle

**Figure 2:**
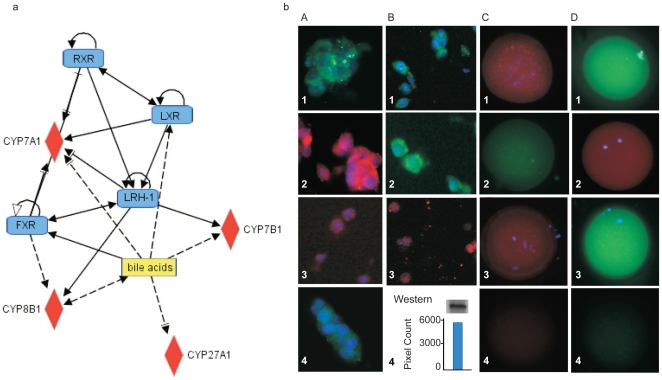
Presence of enzymes and receptors regulating the classical and alternative pathways of bile acid synthesis. In the cytosol of granulosa cells, specific positive staining was registered for CYP7A1 (Panel A, image 1), CYP27A1 (Panel A, image 2), and CYP7B1 (Panel A, image 3). Specific positive staining for Inhibin β was used to confirm granulosa cell type (Panel A, image 4). In oocytes, specific positive staining was identified for CYP7A1 (Panel C, image 1), CYP7B1 (Panel C, image 2), and CYP8B1 (Panel C, image 3). The receptors known to regulate the bile acid synthesis pathway were also present in granulosa cells and oocytes. In granulosa cells, specific positive staining was registered for FXR (Panel B, image 1), LRH-1 (Panel B, image 2), and RXRα (Panel B, image 3). Using western blot of granulosa cells, a specific reacting band for the receptor LXRα was observed (Panel B, image 4). In human oocytes, specific positive staining was also demonstrated for the receptors FXR (Panel D, image 1), RXRα (Panel D, image 2), and LRH-1 (Panel D, image 3). Secondary antibody controls are presented in images 4 of Panels C and D.

Initially, we applied gene chip array to search for the presence of mRNA transcripts of these enzymes in granulosa cells from 10 patients. The assay identified the presence of mRNA for the key enzymes in the classical and the alternative pathways of bile acid synthesis: CYP7A1, CYP27A1, CYP7B1, and CYP8B1 (Figure 2a). Next, the presence of these genes was verified at the protein level by immunofluorescence (IF) with specific antibodies. Granulosa cells from four different patients were analyzed for the presence of CYP7A1, CYP27A1, CYP7B1, and CYP8B1. Specific positive staining was registered for enzymes from both the classical and alternative pathways of bile acid synthesis, including CYP7A1, the rate-limiting enzyme in the classical pathway, CYP27A1, the first and rate-limiting enzyme of the alternative pathway, and CYP7B1 from the alternative pathway of bile acid synthesis (Figure 2b, panel A). The enzyme CYP8B1 was not detected in granulosa cells. Although the result was negative, it served as IF specificity control (not shown).

It is possible that the oocytes which reside within the ovarian follicle also participate in the synthesis of bile acids. We analyzed discarded, non-fertilized human oocytes by IF with specific antibodies for the key enzymes in both bile acid synthesis pathways. We identified specific positive staining of CYP7A1, CYP7B1, and CYP8B1 (Figure 2b, panel C). The immunostaining for CYP27A1 from the alternative pathway was negative (not shown). Bile acid enzyme data from IF in granulosa cells and oocytes are summarized in Table 1.

**Table 1:** Presence of bile acids synthesis enzymes in human ovarian follicle.

Classical Pathway Enzyme	Granulosa Cells	Oocytes
CYP7A1	present	present
CYP8B1	absent	present
CYP27A1	present	absent
**Alternative Pathway Enzyme**		
CYP27A1	present	absent
CYP7B1	present	present

Protein presence was analyzed with enzyme specific antibodies and immunofluorescence. Present - the enzyme is present at a protein level; absent - the enzyme is not present at a protein level.

### The Bile Acid Synthesis Pathway is Functional in the Ovarian Follicle

**Figure 3:**
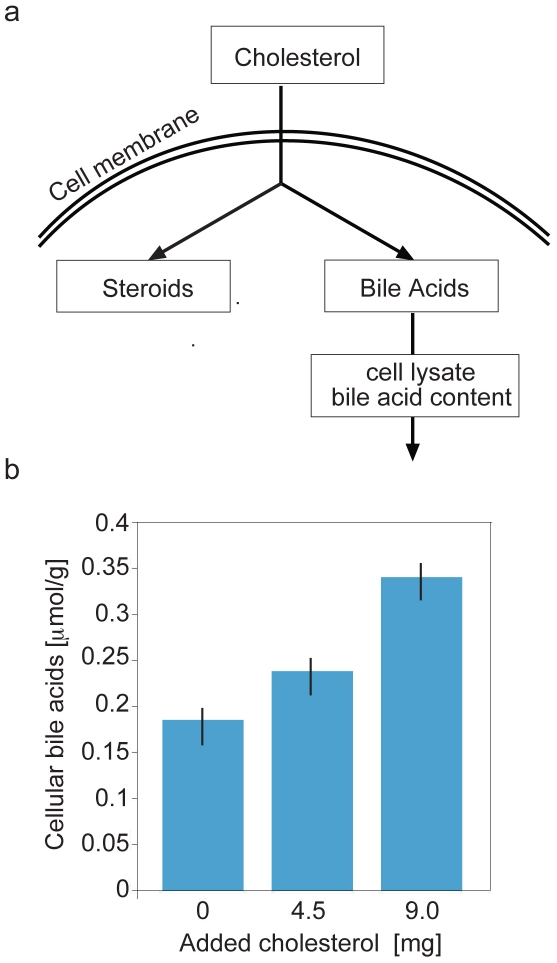
Bile acid production in primary cultured human cumulus granulosa cells (CGC). Cholesterol entering granulosa cells may be used to form sex steroids or bile acids (Panel a). With increasing cholesterol concentration in media, CGC demonstrate dose-dependent bile acid content (Panel b).

To test for bile acids synthesis in granulosa cells we cultured primary human cumulus granulosa cells (CGC) and exposed them to media containing different cholesterol concentrations (13.95mg/dL, 26.09mg/dL, 46.15mg/dL) loaded in human low density lipoprotein (LDL) particles. After 48 hours culture medium and cells have been analyzed for presence of bile acids at time 0 and time 48 hours. The experiment was repeated in triplicate. We measured a CGC-specific dose-dependent increase in production of bile acids in response to increasing cholesterol in CGC media (Figure 3). The results of this experiment clearly demonstrate that CGC respond to changes in cholesterol concentration by synthesizing bile acids. This suggests that the enzymes which we have identified to be present in the granulosa cells are part of a functional pathway in these cells. The presence of all major constituents of the classical pathway, including elements never found outside the liver is curious and novel. The relative contributions of the classical and alternative pathways to the observed bile acid synthesis remains to be determined.

### Receptors Known to Regulate the Bile Acid Synthesis Pathway are Present in the Ovarian Follicle

The bile acid synthesis pathway is known to be regulated by nuclear receptors. We examined primary human granulosa cells and discarded, non-fertilized oocytes for presence of FXR, RXRα, LXRα, and LRH-1 by protein-specific antibodies and IF. FXR, which is known to have as its ligand bile acids, was found to be present in the cytosol of granulosa cells (Figure 2, panel B) as well as in the oocytes (Figure 2, panel D) [11]–[13]. To our knowledge, this is the first report of this receptor in the human ovary. Furthermore, by staining granulosa cells and oocytes with RXRα and LRH-1 specific antibodies we observed specific positive staining for both receptors in three independent experiments with cells from three different patients (Figure 2, panels B and D). The receptor LXRα has never been previously described in the human ovary [6], [23]–[25]. By applying western blot (WB) with LXRα specific antibodies we consistently observed specifically reacting 64kDa band that correspond to LXRα in granulosa cell lysates from four different healthy patients (Figure 2, panel B). Data for presence of receptors from the bile acids pathways are summarized in Table 2.

**Table 2:** Presence of receptors known to regulate the bile acids synthesis pathways.

Receptor	Granulosa Cells	Oocytes
FXR	present, IF	present, IF
RXR alpha	present, IF	present, IF
LRH1	present, IF	present, IF
LXR alpha	present, WB	absent

Protein presence was analyzed with receptor specific antibodies and either immunofluorescence (IF) or western blot (WB). (Present) – the receptor is present at a protein level; (absent) – the receptor is not present at a protein level.

## Discussion

Our results strongly argue for the presence of all of the components of the bile acid synthesis pathway, including the key enzymes in bile acid synthesis as well as the receptors that regulate expression of these enzymes, and the end product bile acids. To our knowledge, this is the first report of this pathway in the human ovary.

Although it is widely accepted that the classical pathway of bile acid synthesis is present only in the liver, our data clearly suggests that the key enzymes from both the classical and alternative synthesis pathways are present in the ovarian granulosa cells and oocytes. To our knowledge, these findings are the first demonstration for presence of CYP7A1, CYP27A1, CYP7B1, and CYP8B1 at both mRNA and protein level in granulosa cells. This is also the first demonstration of the presence of these enzymes in the oocytes as proteins. CYP7A1 is considered to be a liver-specific enzyme, although it has been reported to be present in human prostate [6]. CYP27A1 has been identified in a wide range of extrahepatic tissues, but has not been previously reported in the human ovary [26]. CYP8B1 has only been identified in the liver prior to our experimental results [27]. CYP7B1 is known to have wide tissue distribution, but has not been previously identified in the human ovarian follicle [9]. The presence of bile acids and the key enzymes of both pathways in ovarian follicles suggests for the presence of a fully functional ovarian pathway locally producing bile acids. Presently it is not clear whether bile acids have been synthesized mainly by the granulosa cells, oocytes, or both.

Our results suggest that granulosa cells have the key enzymes to synthesize bile acids from cholesterol, and that this is an active functional pathway. The balance between cholesterol use in steroidogenesis and degradation in the bile acid pathway is not known, but most likely there is a fine balance which may prove crucial to oocyte maturation. In support of this hypothesis are recently published experimental data with rodents where a role for LRH-1 in steroidogenesis, gonadal development, aromatase expression, and progesterone biosynthesis was clearly documented [17], [28]. Another recent study has linked LRH-1 with ovulation in a rodent model, demonstrating that this receptor is required for rodent ovarian follicular development and fertility [17].

Given that the ovarian follicle is a highly privileged environment, it is likely that bile acids play a specific role beyond simply acting as detergents and carriers of cholesterol. Full characterization of the follicular-specific role of bile acids remains to be determined.

## Methods

### Ethics statement

Follicular fluid, granulosa cells and blood serum were obtained under Beth Israel Deaconess Medical Center (BIDMC) IRB approval (Protocol #2003-P-000289) after appropriate written informed consent from women undergoing in IVF. Discarded, non-fertilized metaphase II human oocytes were obtained under BIDMC IRB approval with waiver of informed consent from IVF cycles in which oocytes failed to fertilize and would have otherwise been discarded. Waiver of informed consent for discarded, non-fertilized oocytes was granted by BIDMC IRB because no patient-identifying information was obtained with the pooled, anonymous samples of discarded tissue, and no protected health information was used.

### Follicular fluid, granulosa cell, serum, and discarded, non-fertilized oocyte collection and processing

All follicular fluid from large (mean diameter >16mm) follicles, granulosa cells and blood plasma retrievals have been performed at the Boston IVF Surgery Center of Waltham, MA as previously described [29]. Discarded, non-fertilized metaphase II human oocytes were obtained under BIDMC IRB approval with waiver of informed consent from IVF cycles in which oocytes failed to fertilize and would have otherwise been discarded. Oocytes were fixed in 4% paraformaldehyde for 20 minutes at RT and stored at 4°C for immunofluorescent analyses.

### Total bile acid assays

Total bile acid concentration in follicular fluid and cell culture media was determined using the BioQuant Total Bile Acids Assay Kit (Enzymatic Cycling, San Diego, CA) as recommended by the supplier. All assays have been repeated in duplicate. Statistical comparisons were made using Microsoft Office Excel 2003 SP2 version 5.1.2600 (Redmond, WA) with p value <0.05 considered statistically significant.

### Western blot and Immunofluorescence

Standard technique has been used for western blotting and immunofluorescence [29]. The primary antibodies that have been used are: monoclonal mouse anti-human Farnesoid X receptor (Lifespan Biosciences), polyclonal rabbit anti-human Retinoid X receptor α (Santa Cruz), polyclonal rabbit anti-human Liver X Receptor α (Abcam), monoclonal mouse anti-human Liver Receptor Homolog 1 (R+D Systems), polyclonal goat anti-human CYP7A1 (Santa Cruz), polyclonal goat anti-human CYP7B1 (Santa Cruz), polyclonal goat anti-human CYP8B1 (Santa Cruz), polyclonal goat anti-human CYP27A1 (Santa Cruz), and polyclonal goat anti-human Inhibin β (Santa Cruz).

### Granulosa cell culture and bile acids content analysis

Primary granulosa cells (passage 4 or less) have been cultured in six wells plates to 70% confluence in DMEM, 10% fetal calf serum (FCS), 2.5 ng/mL bFGF (Sigma Aldrich), and 25 µg/mL Amphotericin B. Cholesterol (Sigma) was added to the cell culture medium as a LDL complex in the following concentrations: 13.95mg/dL, 26.09mg/dL, 46.15mg/dL. After 48 hours incubation with cholesterol, the culture medium from each well has been removed and stored for cholesterol and bile acids content assay. Cells have been also collected to measure the protein content in the individual wells and use it as a base for comparison. Incubation with LDL alone without cholesterol addition has been used as negative control. All measurements have been performed in duplicates and in three independent experiments with different preparations of primary cell cultures. The cholesterol content in the culture mediums was measured by the Cholesterol Liquid Color Assay (StanBio, Boerne TX) as recommended by the supplier. BioQuant Total Bile Acids Assay Kit (San Diego, CA) was used to determine the bile acids content. After removing the culture medium the total cellular protein amount in each well was measured by the Lawry method (Bio-Rad kit) and used as a base for comparison.

### Granulosa cell gene expression profiling

Pooled GC RNA from 10 patients was analyzed for gene expression profile using Agilent gene chip array. Ingenuity Systems Pathway Analysis Software (Redwood City, CA) was used to interpret microchip array data.
